# Sexy tools: Individual differences in drumming tool shape

**DOI:** 10.3758/s13420-023-00620-1

**Published:** 2023-12-18

**Authors:** Barbara C. Klump

**Affiliations:** 1 Department of Behavioral and Cognitive Biology, University of Vienna, Djerassiplatz 1, 1030 Vienna, Austria; 2 Max Planck Institute of Animal Behavior, Am Obstberg 1, 78315 Radolfzell am Bodensee, Germany; 3 Vienna Cognitive Science Hub, University of Vienna, Kolingasse 14-16, 1090 Vienna, Austria

## Abstract

Heinsohn et al. *Proceedings of the Royal Society B, 290*, 2023.1271, (2023) report that the choice of tool type (drumsticks or seed pods) and the shape of drumsticks manufactured by palm cockatoos differ among individuals. This variation does not seem to be culturally transmitted as no spatial correlation between proximity of display trees and tool shape was found.

## Main text

Palm cockatoos (*Probosciger aterrimus*) stand out among the few parrot species that use tools. In northern Australia, the males manufacture two types of drumming tools from plant material: drumsticks and seed pods. As part of their mating display, they rhythmically beat the tool against a hollow, with individually consistent beat patterns (Heinsohn et al., 2017).

While most studies on individual variation in tool-oriented behaviours investigate differences in tool manufacture and use, Heinsohn et al. (2023) focus on the use of different tool types and tool morphology, thereby filling an important knowledge gap and doing so in a species that stands out as using tools in a sexual rather than the more common foraging context. By assessing over 250 drumming tools collected from 70 display trees, they found that most males only use drumsticks, while few used both types and only a single male preferred seed pods.

The manufacturing process of drumsticks seems stereotyped, with a male first breaking off the leafy end of a branch, then snipping off all side branches, before releasing the tool by snipping it off the branch (Heinsohn et al., 2023). While further detailed observations might reveal unexpected variation in the manufacturing process (Klump et al., 2015), which can result in tool-shape variation (Sugasawa et al., 2017), the possibility of an individual ‘signature’ in the shape of the tool, rather than the way it is made, is intriguing as it suggests that palm cockatoos have a mental image of the tool they want to manufacture. Heinsohn et al. (2023) collected several morphometric measurements for all tools to investigate differences in tool shape variation. For seed pods, they measured: length, width and breadth, and for drumsticks: length, width, chord (shortest distance between both ends of the stick), dry mass, the number of protruding stubs (where birds had snipped off smaller branches during manufacture) and curvature. They found individual consistency in drumstick length and chord and additionally in width and mass when only considering tools found in a single season (seed pods were not analysed due to the low sample size).

Palm cockatoos don’t seem to be limited by raw materials to make their drumming tools, suggesting that consistency in tool shape within individuals is the result of the manufacture itself (Heinsohn et al., 2023). This said, the influence of raw material properties is an important factor to consider when quantifying differences in the morphometry of tools because features of the raw material prevent certain behavioural actions from being performed during manufacture (Klump et al., 2015). For example, even the powerful and large bills of palm cockatoos will reach their limits when a branch reaches a certain diameter.

Interestingly, the morphometric aspects of drumstick tools in palm cockatoos differ in their level of potential alteration and modification. Some aspects (especially the length of the tool) can be altered by the cockatoo during tool manufacture (by snipping off the branch at a certain point). Other aspects can only be influenced indirectly (chord, dry mass, number of protruding stubs – through the selection of the tool length), while yet other aspects are predetermined by the raw material properties and have to be selected before the start of manufacture (width, curvature).

The influence of raw material properties on the shape of the resulting tool is well studied in humans (for examples see Klump et al., 2015), highlighting that both material constraints and the skill of the tool maker are important to consider when assessing variation in tool shape. Wild-caught New Caledonian crows (*Corvus moneduloides*) show individually consistent variation in how they make hooked stick tools (used to extract embedded food like grubs and larvae from deadwood). However, part of this variation (e.g., manufacture method) is driven by the raw material's properties (Klump et al., 2015). More importantly, both manufacture method and material properties influence tool morphology in this species (Sugasawa et al., 2017). While individual differences at the level of the tool shape were not investigated in New Caledonian crows, analyses across individuals showed that small changes in tool shape can strongly affect the suitability and efficacy of a tool (Sugasawa et al., 2017). If this were true in palm cockatoos, the individually consistent variation in tool shape might also affect the sound they produce when used for drumming.

Inter-individual variation in behaviour is usually the result of several factors including genetic predisposition, ecological differences, individual trial-and-error learning, and social learning. As the availability of branches from which drumsticks could be manufactured was the same across the study sites, ruling out ecological differences, Heinsohn et al. (2023) conducted a spatial analysis to investigate the possibility of social learning of tool shapes. Morphometric measurements of drumsticks (from different trees) were, however, not more similar when found at trees in close proximity. This lack of evidence supporting social learning is not surprising as palm cockatoos are rather solitary, with limited opportunities to observe non-related males manufacturing and using tools. Given the long developmental period of young, there is potential for vertical transmission of skills (Heinsohn et al., 2023). However, young usually disperse before they mature, making it unreasonable to assume similarity in tool designs in neighbouring displaying males.

Heinsohn et al. (2023) put tool shape variation back on the tool-use research landscape. Their study highlights the importance and value of studying animal artefacts, especially in systems where marking a large number of wild individuals and/or conducting controlled experiments in (short-term) captivity is challenging due to ethical and/or logistical constraints.

Yet, despite the many strengths of this paper, the devil is in the detail: Key to determining individual differences is the reliable identification of individuals and the ability to match tools to their maker. While the logistical constraints with regard to individual identification in palm cockatoos are evident, one aspect is worth exploring in a bit more detail, as its potential implications are far-reaching. While the terminological difference between hollow, display site and display tree is at times unclear, Heinsohn et al. (2023) assessed drumstick shape per display site where at least six tools had been found (*n =* 12), with tools found under a given tree assumed to be from the same male. For the spatial analysis, however, all display trees were included (*n=*70), and some of them were in close proximity (analysed in groups of trees within 50 m or 100 m). As male palm cockatoos display on multiple trees within their territory, in a previous study investigating vocal complexity in this species (Zdenek et al., 2015), such short distances (< 2 km) between display trees would have been classified as belonging to the same territory, suggesting that it is possible that not every single display tree in the current study was used by a different male.

While purely speculative without further information on how display sites were assigned to a given male, the spatial context and a reliable way to identify individual males, the consequences of imagining a scenario where some of the display trees sampled by Heinsohn et al. (2023) belong to the territory of a single male – rather than different males – are intriguing. Under such a scenario, tools collected from different trees are manufactured by the same rather than different individuals and – given the finding that tools collected from neighbouring trees did not show similarities in shape – would suggest that male palm cockatoos manufacture tools of specific shape for a specific display tree. This possibility opens up exciting future research directions. The mating display of palm cockatoos is multi-modal (audiovisual), and sound is produced both by pounding the tool against the hollow and by the male using specific vocalisations. This suggests that the auditory component is an important feature for the female to assess the male’s quality. Palm cockatoos also drum with their feet alone (without a tool), and these sounds appear to be different to the ones produced with a tool (Heinsohn et al., 2023). They also seem to modify tools (at least seed pods) in response to the auditory feedback they get during use. Just imagine if male palm cockatoos not only adjusted the tools they make to a specific tree, but if they did so to account for differences in acoustic properties of hollows in order to achieve a signature sound.
